# Comprehensive Clinical Profile of Mal De Debarquement Syndrome

**DOI:** 10.3389/fneur.2018.00261

**Published:** 2018-05-07

**Authors:** Yoon-Hee Cha, Yong Yan Cui, Robert W. Baloh

**Affiliations:** ^1^Laureate Institute for Brain Research, Tulsa, OK, United States; ^2^School of Community Medicine, University of Tulsa, Tulsa, OK, United States; ^3^Department of Internal Medicine, Columbia Medical Center, New York, NY, United States; ^4^Department of Neurology, David Geffen School of Medicine, University of California, Los Angeles, Los Angeles, CA, United States

**Keywords:** Mal de Debarquement Syndrome, rocking vertigo, persistent postural perceptual dizziness, clinical spectrum, therapeutics

## Abstract

**Background:**

There has been increasing awareness that post-motion triggered rocking self-vertigo can last for months or years, a disorder known as Mal de Debarquement Syndrome (MdDS). A similar feeling of oscillating self-motion can occur without a motion trigger in some individuals, leading to controversy about whether motion triggered (MT) and non-motion triggered (non-MT) symptoms ultimately represent the same disorder. Recognizing the similarities and differences between MT and non-MT MdDS can prevent unnecessary diagnostic testing and lead to earlier and more effective treatments.

**Methods:**

Standardized questionnaire assessment and follow-up interviews of individuals with persistent MT or non-MT MdDS (>1 month) examined at a University Dizziness Clinic.

**Findings:**

Questionnaires were available on 80 individuals with persistent MT MdDS and 42 with non-MT MdDS. Sex distribution (81% female) and age of onset (mean 43.4 ± 12.2 years MT; 42.1 ± 15.2 years non-MT) were comparable between MT and non-MT MdDS (*p* > 0.05). Mean duration of illness was significantly longer in the non-MT group (82.8 ± 64.2 months) than the MT group (35.4 ± 46.4 months) (*p* < 0.001). There was no correlation between trigger type and age of onset or duration of illness for MT MdDS. Improvement with re-exposure to motion (driving) was typical for both (MT = 89%, non-MT = 64%), but non-MT individuals more frequently had symptoms exacerbated with motion (MT = 0%; non-MT = 10%). Peri-menstrual and menstrual worsening of symptoms was typical in both MT and non-MT MdDS (each 71%). Both MT and non-MT MdDS exhibited a higher population baseline prevalence of migraine (23% and 38%, respectively). Benzodiazepines and SSRI/SNRIs were helpful in both subtypes of MdDS (>50% individuals with a positive response). Physical therapy was modestly helpful in the MT (56%) subtype but not in non-MT (15%). Vestibular therapy made as many individuals worse as better in MT and none improved in the non-MT group.

**Conclusion:**

General demographic characteristics and exacerbating factors are similar in MT and non-MT MdDS, but there are differences in the duration of illness, effect of motion on symptoms, and response to therapy. Recognizing clinical features of MdDS subtypes may allow for better tailoring of therapy and potentially serve as classification criteria for new clinical designations.

## Introduction

Historical references to the phenomenon of landsickness date back to the seventeenth century, but recognition of a clinical entity characterized by prolonged landsickness, “Mal de Debarquement Syndrome,” (MdDS), has only occurred in the second half of the twentieth century (1–5). The post-motion triggered feeling of “rocking” as if one was “still on the boat,” that follows sea, air, or land-based motion is a common experience in otherwise healthy individuals, occurring with a prevalence of about 70% (6–10). These short bouts of motion triggered (MT) symptoms last for two or fewer days and are referred to as “landsickness.” In some cases, however, the symptoms can persist for months or years, putting the syndrome into the category of MdDS (11, 12).

The International Classification of Vestibular Disorders defined vertigo as the “sensation of self-motion when no self-motion is occurring or the sensation of distorted self-motion during an otherwise normal head movement.” In this broad sense, the experience of MdDS is a form of vertigo (13). However, it is not vertigo in the intuitive sense of a perception of rotation. MdDS vertigo is oscillating or periodic and is present at rest without head movement. It is described as a rocking, bobbing, or swaying sensation and never as spinning (3, 14).

Recognition of MdDS as a clinical entity appears to be limited. A typical patient with MdDS sees over a dozen health care providers before arriving at a diagnosis (12, 15). The burden of illness of MdDS has been measured as being comparable to multiple sclerosis and as expensive as migraine (15). There are no clinical tests like brain imaging or vestibular function testing that can diagnose MdDS, as these are overwhelmingly normal (11, 16). MdDS has typically been diagnosed by the appropriate clinical history of oscillating vertigo, i.e., rocking, bobbing, swaying, that starts after a motion trigger has ended. Most cases are reported to improve temporarily with re-exposure to passive motion, such as driving a car or getting back on the boat. In this regard, it should not be confused with motion sickness in which the predominant symptom is nausea which occurs during the motion exposure itself (17, 18).

Caring for patients with MdDS represents a challenge for both primary care providers and vestibular specialists because of limited awareness of the spectrum of clinical features that comprise this syndrome as well as the paucity of treatment options. Dizziness and vertigo in general are such common symptoms that clinically distinct though less prevalent disorders such as MdDS might be unrecognized or be considered as “atypical” variants of more common entities such as Meniere’s disease or benign paroxysmal positional vertigo (BPPV) (19–22). Therefore, recognition of the core clinical features of MdDS is critical for diagnosis. When the classical history of rocking vertigo that starts after exposure to passive motion is encountered in a patient, the need for extensive structural brain and inner ear testing is of low yield (16, 21). Moreover, efficient diagnosis and treatment can be critical in reducing the development of co-morbid mood or anxiety disorders and social stigma in MdDS (23).

Over the course of many years of seeing patients with MdDS and performing clinical trials in MdDS, a database of patients and research participants in MdDS studies was created at the University of California, Los Angeles. We had first sampled this database to report on clinical features of MdDS in 2008 and again in 2013 with respect to the association between MdDS and migraine (16, 24). Since then, we have added additional participants as well as obtained data on more nuanced features of the disorder, such as the effect of hormonal cycles, body position, past psychiatric illness diagnoses, circumstances surrounding the onset of MdDS, and effect on employment. These clinical features are provided in this study along with additional data on the efficacy of therapies that individuals with MdDS had tried in the course of their clinical care.

We use two designations for MdDS in this study. Classical motion triggered MdDS, which we refer to as “MT” here, will be defined by the onset of rocking vertigo within 48-h of disembarking from a moving vessel, be it water, air, or land-based motion. Rocking vertigo can start without a motion trigger, however. These patients have presented a unique challenge, since they share nearly all of the features of classical MdDS from the patients’ experience standpoint, but present without an identified motion trigger. In the past, these individuals have been referred to as “mixed” MdDS, since they often have a prior history of MT MdDS, or as “spontaneous” MdDS to denote the non-motion triggered (non-MT) onset (12, 16). Whether non-MT rocking vertigo, which we refer to as “non-MT,” is fundamentally different from MT feelings of rocking vertigo is not known. However, there have been suggestions that the association with migraine and response to certain therapeutic maneuvers may be different in the two entities (16, 24, 25). Therefore, the designation of non-MT MdDS appears to be due to factors other than simple recall bias of a triggering event.

In our current assessment of individuals with MdDS, we have included both MT and non-MT variants in order to present a spectrum of how rocking vertigo may present to either the primary care physician or specialist. Detailed clinical features that may serve as risk factor identification or that may lead to disease sub-typing are presented. Greater recognition of the spectrum of MdDS may guide initial treatment and referral decisions.

## Materials and Methods

The study was carried out in accordance with the approval and recommendations of the UCLA School of Medicine Medical IRB #3. Participants gave written informed consent in accordance with the Declaration of Helsinki (26). Outpatient visits to the neurotology clinic that had been tabulated in a clinical database since 1985 were reviewed for patients with a diagnosis of MdDS.

Criteria for the diagnosis of MdDS included the following: In the case of MT MdDS, criteria included the experience of persistent rocking vertigo that started within 48-h of disembarking from a water, air, or land-based vessel and that lasted at least 1-month. In the case of non-MT MdDS, symptoms had to start without any significant travel within the month before the onset. Significant travel was defined as passive motion exposure lasting at least 2 h. This duration was chosen because it is a length of time that is outside of normal exposures that would likely be experienced by the participant during their daily activities.

Since we were examining the clinical spectrum of rocking vertigo, we did not make restrictions on alleviating or exacerbating factors, such as the effect on re-exposure to passive motion. MT and non-MT were designated strictly on the identification of a proximate motion trigger. All participants in this study had been previously diagnosed with MdDS by an experienced neurotologist (RWB or YHC), without any other cause for symptoms determined after appropriate testing for peripheral or central vestibular dysfunction.

A standardized questionnaire packet and consent form was mailed to the most recent address on file for the participant with a stamped return envelope included. If no response was received within 2 months, a second packet was sent. Each packet contained a consent form to be returned with the questionnaire as well as a “do not wish to participate” card that could be returned to stop any future contact from the study. Therefore, all data tabulated in this study were from individuals who provided written informed consent.

Individuals who had consented to participate were contacted by phone if any answer on a questionnaire was ambiguous. However, not all participants with incomplete data could be contacted. Responses were scored if they were clear and unambiguous, so different numbers of responses were available for each set of questions. All percentages were rounded to the nearest whole number.

### Statistics

The following statistical analyses were performed: (1) for continuous variables, such as age, a two sample *t*-test of means with a two-tailed *p*-value threshold of 0.05 for significance; (2) for categorical variables with more than five counts, a Chi-squared test; (3) for categorical variables with less than five counts, a Fisher’s exact test; (4) for age of onset and duration for MT MdDS, a one-way ANOVA with a *post hoc* correction for significance; and (5) for the effect of activities on symptoms, we converted responses of “better,” “same,” and “worse” into percentages, and assigned “better” as +1, “same” as 0, and “worse” as −1, and performed ordered logistic regression to determine the odds of favoring one category vs. another.

## Results

A total of 283 questionnaires were mailed to patients diagnosed with MdDS. A total of 135 questionnaires/consent forms were returned. Twenty-two additional packets were returned by the post office because the individuals were no longer at the address on file. Eleven “do not wish to participate” cards were returned resulting in an approximately 52% response rate. Of the 135 returned packets, 132 were complete enough for analysis; an additional 10 were removed after secondary review because the respondents did not have an MdDS episode lasting at least 1 month. Data from the resulting 122 participants were analyzed. If a participant had experienced both MT and non-MT episodes, they were categorized according to their last episode, yielding 80 MT and 42 non-MT designations.

Basic demographic features, such as sex, age of onset, handedness, race, and duration of illness are provided in Table 1.

**Table 1:** Basic demographic characteristics.

	Total	Motion triggered (MT)	Non-MT	*p*-Value
		
	Total (%) *n* = 120	Total (%) *n* = 80	Total (%) *n* = 40	
Sex (F:M)^a^	99:23 (81:19%)	65:15 (81:19%)	34:8 (81:19%)	
**Handedness**				
Right	107 (89%)	70 (88%)	37 (93%)	0.5403
Left	11 (9%)	9 (11%)	2 (5%)	
Ambidextrous	2 (2%)	1 (1%)	1 (3%)	
**Race/ethnicity**				
White	104 (87%)	71 (89%)	33 (83%)	0.8794
Black	0 (0%)	0 (0%)	0 (0%)	
Asian	5 (4%)	2 (3%)	3 (8%)	
Other/mixed	10 (8%)	6 (8%)	4 (10%)	
Hispanic	9 (8%)	8 (10%)	1 (3%)	
**Age of onset first episode (years)**				
Mean (SD)		43.4 (12.2)	42.1 (15.2)	0.6138
Median		44	42	
Range		12–70	16–71	
**Duration of symptoms (months)**				
Mean (SD)		35.4 (46.4)	82.8 (64.2)	<0.0001
Median		12	62.9	
Range		1–204	18–255	

*^a^Total based on 42 non-MT*.

There was a similar distribution of women vs. men in the MT vs. non-MT groups with a strong skew toward women (81% women vs. 19% men). The percentage of left-handed individuals was similar to population baselines (27). Of the respondents, the racial identification showed a higher proportion of self-identified whites, lower blacks, comparable Asians, and lower Hispanics compared to the latest general US census available from 2016 [76.9% whites, 13.3% black, 5.7% Asian, mixed 2.6%, and Hispanic 17.8%] (https://www.census.gov). Though both the mean and median age of onset of symptoms were earlier in the non-MT group, the difference was not significant. The main clear difference between the MT and non-MT groups was the longer duration of illness in the non-MT group [MT mean 35.4 (46.4), non-MT mean 82.8 (64.2), *p* < 0.0001]. Consistent with the difference in duration, 75% (60 out of 80) of MT MdDS and 90% (38 out of 42) of non-MT MdDS participants were continuing to experience symptoms at the time of the questionnaire.

The percentage of water, air, or land-based triggers, the age of onset of symptoms, and the duration of episodes experienced to date for each trigger were assessed for the MT MdDS group (Table 2). Since some episodes of MdDS were triggered by a combination of these exposures, the sum of the percentages was greater than 100%. Water-based triggers were the most common (69%), followed by air (33%), and then land (11%). One-way ANOVA with one factor (duration) and three levels (triggers), showed no significant effect of trigger type on duration (*f* ratio = 1.01, *p* = 0.367). Age of onset accounted for very little of the variance in duration of illness, *R*^2^ = 0.0257. Similarly, there was no significant difference between age of onset and trigger type (*f* ratio = 1.02, *p* = 0.364).

**Table 2:** Motion triggers for motion triggered Mal de Debarquement Syndrome.

		Age of onset (years)				Duration (months)			
Trigger	Total *n* = 80	Mean	SD	Median	Range	Mean	SD	Median	Range
Any water	55 (69%)	44.5	11.6	44.0	21–70	31.2	39.7	12.0	1–156
Any air	26 (33%)	46.3	7.0	46.0	34–57	50.1	49.2	36.0	1–144
Any land	9 (11%)	34.6	11.6	32.0	24–59	43.0	56.3	11.5	1–204
All		43.6	11.6	44.0	21–70	35.4	46.4	12.0	1–204

Modulation of the rocking perception by re-exposure to passive motion, especially driving, or with walking, standing, lying down, or closing the eyes was evaluated on a basic scale of whether the rocking was better (decreased), stayed the same, or worsened (increased) in each of these body positions (Table 3).

**Table 3:** Modulating feature of motion perception.

	Motion triggered (MT)	Non-MT	*p*-Value
		
Action	Total (%) *n* = 80^a^	Total (%) *n* = 42^a^	
**Driving**			
Better	71 (89%)	27 (68%)	0.9980
Same	8 (10%)	9 (23%)	
Worse	1 (1%)	4 (10%)	
**Walking**			
Better	29 (37%)	10 (24%)	0.2020
Same	21 (27%)	15 (37%)	
Worse	29 (37%)	16 (39%)	
**Standing**			
Better	10 (13%)	4 (10%)	0.2580
Same	31 (39%)	19 (46%)	
Worse	39 (49%)	18 (44%)	
**Lying down**			
Better	43 (54%)	11 (27%)	0.2750
Same	16 (20%)	19 (46%)	
Worse	21 (26%)	11 (27%)	
**Eyes closed**			
Better	11 (14%)	4 (10%)	0.2750
Same	32 (40%)	18 (44%)	
Worse	38 (47%)	19 (46%)	

*^a^Percentages based on the number of respondents for each situation*.

Respondents with MT MdDS were nearly always better when driving and rarely worse (89% better vs. 1% worse). The majority of individuals with non-MT MdDS also improved with driving, but not to as great an extent (64%), with a minority feeling worse (10%). In general, the effect of walking was as likely to make symptoms worse as better (both 37%) in the MT group. However, standing still was clearly more likely to worsen symptoms than to make them better (49% worse vs. 13% better). Lying down was more frequently reported as reducing the feeling of motion in MT MdDS than in non-MT MdDS (54 vs. 26%), but there was an important fraction of respondents in whom the rocking perception actually increased when recumbent (26%) in both groups. Removing vision was more likely to make symptoms worse than to make them better in both groups.

A history of migraine headaches, episodic vertigo, and any prior mental health diagnoses were specifically queried because of their known association with chronic dizziness (18, 28, 29) (Table 4). Respondents also reported about the context of their symptom onset, such as whether they were at a normal baseline or were experiencing any number of stressors at the onset of their symptoms (Table 4). Examples of physical stressors were concurrent medical illness or recent surgery. Mental health stressors included situations such as increased job or family stress or financial problems. Sleep deprivation was generally over a period of several weeks. Medication changes were of any variety, but the most important was rapid tapering of antidepressants. Hormone changes were typically abrupt switching of oral contraception. *P*-values reflect differences between the MT and non-MT group totals. There were no significant differences in past medical history or context of trigger between the MT and non-MT groups, but there was a trend toward a higher rater of baseline migraine in the non-MT group.

**Table 4:** Associated diagnoses and conditions.

	Motion triggered (MT)			Non-MT			*p*-Value
	Total (%) *n* = 80	Women (%) *n* = 65	Men (%) *n* = 15	Total (%) *n* = 42	Women (%) *n* = 34	Men (%) *n* = 8	
**Past history**							
Migraine	18 (23%)	15 (23%)	3 (20%)	16 (38%)	16 (47%)	0 (0%)	0.1068
Episodic vertigo	6 (8%)	3 (5%)	3 (20%)	5 (12%)	3 (9%)	2 (25%)	0.6664
Major depressive disorder	26 (33%)	19 (29%)	7 (47%)	11 (26%)	10 (29%)	1 (13%)	0.6079
Bipolar disorder	0 (0%)	0 (0%)	0 (0%)	0 (0%)	0 (0%)	0 (0%)	1.0000
Generalized anxiety disorder	17 (21%)	13 (20%)	4 (27%)	12 (29%)	11 (32%)	1 (13%)	0.4973
Panic disorder	10 (13%)	7 (11%)	3 (7%)	10 (24%)	9 (26%)	1 (13%)	0.1784
Obsessive compulsive disorder	2 (3%)	1 (2%)	1 (7%)	0 (0%)	0 (0%)	0 (0%)	0.7773
Post-traumatic stress disorder	2 (3%)	2 (3%)	0 (0%)	3 (7%)	2 (6%)	1 (13%)	0.4542
Attention deficit disorder	1 (1%)	1 (2%)	0 (0%)	0 (0%)	0 (0%)	0 (0%)	0.4669
**Condition at onset**							
Normal baseline	29 (36%)	22 (34%)	7 (47%)	20 (48%)	17 (50%)	3 (38%)	0.3064
Physical stress	28 (35%)	21 (32%)	7 (47%)	13 (31%)	9 (26%)	4 (50%)	0.8041
Emotional stress	37 (46%)	29 (45%)	8 (53%)	18 (43%)	15 (44%)	3 (38%)	0.8679
Sleep deprivation	20 (25%)	18 (28%)	2 (13%)	14 (33%)	11 (32%)	3 (38%)	0.4455
Medication change	4 (5%)	4 (6%)	0 (0%)	5 (12%)	3 (9%)	2 (25%)	0.3069
Hormone change	9 (11%)	9 (14%)	0 (0%)	7 (17%)	7 (21%)	0 (0%)	0.5756
Combination of > = 2	37 (46%)	30 (46%)	7 (47%)	20 (48%)	15 (44%)	5 (63%)	0.8855

In addition, the presence of current anxiety or depression was determined by completion of the hospital anxiety and depression scale (HADS) (Table 5). The HADS is a well-validated self-reported scale of seven items that query anxiety and seven items that query depression symptoms (30). Higher scores indicate more severe symptoms.

**Table 5:** Hospital anxiety and depression scale.

	Motion triggered (MT)	Non-MT	Mean difference	*p*-Value
	*n* = 80	*n* = 39		
**Anxiety**				
Raw score (SD)	7.69 (4.29)	8.82 (3.89)	1.13	0.1673
Normal < = 7	30 (49%)	16 (41%)		
Borderline 8–10	21 (26%)	9 (23%)		
Abnormal 11–21	20 (25%)	14 (36%)		
**Depression**				
Raw score (SD)	5.84 (3.98)	6.77 (4.25)	0.93	0.2443
Normal < = 7	53 (66%)	26 (67%)		
Borderline 8–10	18 (23%)	4 (10%)		
Abnormal 11–21	9 (11%)	9 (23%)		

There were more individuals in the non-MT group whose scores were within the abnormal range for both anxiety (36 vs. 25%) and depression (23 vs. 11%) compared to the MT group, but the mean scores were not different between the two groups statistically.

Since the majority of affected individuals with MdDS were women, the relationship between MdDS and the effect of hormone transitions was queried (Table 6). Ages of onset of menarche, menopause, and MdDS showed no statistically significant differences between the MT and non-MT groups. Peri-menstrual worsening of symptoms was typical (71%) in both groups. Of MdDS occurring during hormonal transitions, only contraception change was significantly more common in the non-MT group.

**Table 6:** Hormone status at onset of Mal de Debarquement Syndrome (MdDS).

	Motion triggered (MT)	Non-MT	*p*-Value
		
	Total (%) *n* = 65	Total (%) *n* = 31	
**Premenstrual MdDS onset**	28 (43%)	14 (45%)	0.8474
Age of menarche years (SD)	12.8 (1.3)	12.6 [1.1]	0.4617
**Effect of menses**			
Sx worse perimenstrual	20 (71%)	10 (71%)	0.6309
Sx better perimenstrual	1 (4%)	1 (7%)	
**Post-menopausal MdDS onset**	37 (57%)	17 (55%)	0.8474
Age of menopause (SD)	47.3 (8.2)	44.8 [8.9]	0.3157
**MdDS onset during hormone transitions**			
Perimenopause^a^	22 (34%)	6 (20%)	0.2223
Pregnancy	2 (3%)	1 (3%)	1.000
Postpartum	2 (3%)	3 (10%)	0.3243
Wean from nursing	2 (3%)	1 (3%)	1.000
Oophorectomy	2 (3%)	1 (3%)	1.000
Contraception change	1 (2%)	4 (13%)	0.0362

*^a^Hot flashes, night sweats, and vaginal dryness*.

Symptom severity was assessed on a 10-point scale in which 1 represented no rocking and 10 represented symptoms so severe that standing was not possible (Appendix). Since women and men could experience MdDS symptoms differently, scores were analyzed separately for women and men as well as for all MT and all non-MT respondents. The median rating of symptom severity by women was 0.5 higher in the MT group and 1 point higher in the non-MT group compared to men (Table 7).

**Table 7:** Symptom severity and effect on employment.

	Motion triggered (MT)			Non-MT		
	Total *n* = 78	Women (%) *n* = 63	Men (%) *n* = 15	Total *n* = 40	Women (%) *n* = 32	Men (%) *n* = 8
**Symptom rating**						
1	3 (4%)	1 (2%)	2 (13%)	1 (3%)	1 (3%)	0 (0%)
2	3 (4%)	3 (5%)	0 (0%)	2 (5%)	0 (0%)	2 (25%)
3	9 (12%)	4 (6%)	5 (33%)	5 (13%)	4 (13%)	1 (13%)
4	12 (15%)	9 (14%)	3 (20%)	5 (13%)	3 (9%)	2 (25%)
5	17 (22%)	14 (22%)	3 (20%)	12 (30%)	10 (31%)	2 (25%)
6	15 (19%)	13 (21%)	2 (13%)	8 (20%)	8 (25%)	0 (0%)
7	7 (9%)	7 (11%)	0 (0%)	3 (8%)	3 (9%)	0 (0%)
8	4 (5%)	4 (6%)	0 (0%)	1 (3%)	0 (0%)	1 (13%)
9	0 (0%)	0 (0%)	0 (0%)	1 (3%)	1 (3%)	0 (0%)
10	0 (0%)	0 (0%)	0 (0%)	0 (0%)	0 (0%)	0 (0%)
Median	4.5	5	3.5	5	5	4
**Employment change due to Mal de Debarquement Syndrome**						
Transferred to part-time	10 (13%)	7 (11%)	3 (20%)	6 (15%)	4 (13%)	2 (25%)
Retired/disabled	13 (17%)	12 (19%)	1 (7%)	9 (23%)	8 (25%)	1 (13%)

Additionally, the effect of MdDS symptoms on transitions to part-time work if the individual was originally working full-time, or to early retirement or disability due to MdDS, was determined separately for women and men. 30% of MT and 38% of non-MT participants reported making a job status change because of their symptoms (Table 7).

Finally, we determined how treatments available to patients with MdDS at the time of the survey were scored. Though some experimental therapies have been investigated for MdDS to date, only therapies that were available through standard medical care were assessed for this study (25, 31–35).

Scores were made on a 1–6 scale with the following descriptors: 1 = greatly relieved symptoms; 2 = moderately improved symptoms; 3 = small but noticeable improvement; 4 = minimal improvement, if any; 5 = no improvement at all; and 6 = made symptoms worse.

Treatments that were tried by at least 10 individuals are listed in Table 8. Treatments that were rated at 1, 2, and 3 represented those that provided unambiguous positive responses and were combined under a single “Response” percentage.

**Table 8:** Therapeutic responses.

	Motion triggered (MT)				Non-MT			
	# participants	Median	Range	Response^a^	# participants	Median	Range	Response^a^
Benzodiazepines	39	2	1–5	34 (87%)	14	1.5	1–6	11 (79%)
SSRI	17	3	1–6	9 (53%)	9	5	1–6	3 (33%)
Mixed SSRI/SNRI	10	3	2–6	6 (60%)	5	2	1–6	3 (60%)
TCA	11	4	1–6	4 (36%)	5	5	2–5	1 (20%)
Antiemetics	23	5	3–6	2 (9%)	10	5	3–6	1 (10%)
Physical therapy	39	3	1–5	22 (56%)	13	5	3–6	2 (15%)
Vestibular therapy	32	4	1–6	12 (38%)	10	5	4–6	0 (0%)
Stress reduction	20	3	1–6	11 (55%)	12	4	2–5	5 (42%)

*SSRI, selective serotonin reuptake inhibitor; SNRI, selective norepinephrine reuptake inhibitor; TCA, tricyclic amine*.

*^a^Score of 1, 2, or 3*.

Median responses to benzodiazepines and mixed SSRI/SNRIs were clearly on the favorable side in the majority of MT and non-MT participants. SSRIs, stress reduction, and physical therapy were reported to be somewhat helpful in the MT group, but less so in the non-MT group. Vestibular therapy could be somewhat helpful in the MT group, but was not helpful in the non-MT group. The median response rate was lower for the non-MT group for all categories of treatment other than benzodiazepines and SSRI/SNRIs. Other treatments tried by fewer than 10 individuals included: acetazolamide, other diuretics, low salt diet, beta-blockers, calcium channel blockers, and anticonvulsants. None were reported to confer any improvement, however.

## Discussion

We report detailed clinical features of MdDS in order to elucidate the spectrum of historical features that may aid in recognizing, diagnosing, and treating individuals who present with rocking vertigo. New information provided through this study include a broad range of demographic data, such as sex, handedness, race, age, and triggers, as well as potentially contributing factors to the onset of symptoms, such as a past history of migraine or homeostatic altering factors, such as stress, medication changes, and sleep deprivation (Tables 1, 2, and 4). We also provide a measure of symptom severity, showing differences between women and men as well as the effect symptoms on employment status (Table 7).

Exacerbating and mitigating effects of body position, motion, and vision are reported. Our study shows that at least a quarter of individuals with MdDS may actually feel worse when they lie down (Table 3). This may be considered unusual for a balance disorder in which an intuitive assumption might be that getting off of one’s feet would be preferred. However, these individuals report that lying still and flat increases the internal motion perception, similar to how a very quiet room enhances the perception of tinnitus. This should not be confused with BPPV, however, which can be triggered by the motion of lying down. The vertigo of BPPV is specifically triggered by vertical head motion, lasts less than 1-min, and resolves once the head stops moving (36). Unlike BPPV, the motion perception of MdDS is persistent and not specifically head movement triggered.

Re-exposure to motion in the context of driving improved symptoms in the majority of participants, but there were more instances of driving worsening the rocking in the non-MT (10%) group compared to the MT group (1%). While 89% of MT MdDS individuals felt specifically better with driving, the percentage was lower (68%) in the non-MT group. Though this was not a statistically significant difference, it does suggest that there may be some underlying pathophysiological differences between the two groups (Table 3).

Though most individuals with MdDS did not meet current criteria for a depression or anxiety, the percentage of abnormal scores on both depression and anxiety subscores of the HADS was higher in the non-MT vs. the MT group. Whether this was simply due to their longer duration of illness is not clear, however (Table 5). There was a relatively high percentage of participants who had had a prior diagnosis of major depressive disorder (overall 33% for MT and 26% for non-MT), which is much higher than the reported population prevalence of 6–15% for depression (37). Similarly, the percentage of participants diagnosed with generalized anxiety disorder was also high at 21% for MT and 29% for non-MT, which is 2–3 times the prevalence of generalized anxiety disorder in the population (38). Since these assessments were of pre-existing mood and anxiety disorders, it is possible that they may have been premorbid risk factors for the development of MdDS. However, recall bias may potentially skew reporting, since current mood and anxiety symptoms may also affect recollection of past diagnoses.

The prevalence of migraine in MdDS patients at 23% MT and 38% non-MT revealed in this study is higher than the US population baseline of roughly 18% in women and 9% in men (Table 4) (39). A more specific evaluation of the association between migraine and MdDS was reported in an earlier study of this database (24). This prior study reported that though the general prevalence of migraine was similar in the two groups, the non-MT group generally began experiencing migraines before the onset of MdDS, while the MT group more frequently developed migraine along with the onset MdDS symptoms (24). Additionally, headache frequency and severity generally increased in both groups with the onset of MdDS symptoms.

An evolving issue is whether MdDS should be considered a form of vestibular migraine. Sensory hypersensitivities can develop as a function of MdDS itself and many of these symptoms overlap with migraine symptoms. For example, chronic nausea can develop in MdDS (16). On this baseline, any moderately severe headache that is worse with movement would automatically meet the criteria for migraine, creating a low bar for diagnosing migraine in an individual who has already developed MdDS (40). This subtlety should be considered when attempting to treat MdDS as a form of vestibular migraine.

Vestibular migraine has been conceptualized as an episodic disorder in which discrete vestibular events occur in the context of a history of migraine, but in which vestibular symptoms do not exceed 72 h (41). Therefore, MdDS would not be considered a form of vestibular migraine under current diagnostic criteria due to its chronicity. However, the pathophysiology could be related and there may be some support for treating MdDS with lifestyle modifications typically used to treat vestibular migraine (42). However, an argument against MdDS as specifically being a form of migraine is that SSRIs are generally much more effective than tricyclic amines in reducing MdDS symptoms (Table 8), while this pattern is exactly opposite the treatment pathway for migraine headaches in which SSRIs have not shown evidence of efficacy greater than placebo or tricyclic amines (43, 44).

The most striking feature of MdDS may be the duration of illness considering that it develops out of what should have been an otherwise self-limiting disorder. The probability of symptoms resolving with or without treatment declines quite rapidly after the first month, with a flattening out of the response curve at 12 months (16). There is also a striking difference in the duration of illness between the MT and non-MT groups, which was consistent with an overall lower response to treatment in the non-MT group. Notably, while both groups reported a high response rate to benzodiazepines, and moderately high levels of response to SSRI/SNRIs, they differed in that both physical and vestibular therapy were rarely reported to be helpful in non-MT MdDS (Table 8). Latent comorbidities, such as migraine, a mood, or anxiety disorder, which were slightly higher in the non-MT group, may be factors that work against symptom resolution (Tables 4 and 5).

An additional consideration is that non-MT MdDS might be diagnosed later than MT MdDS, since there is no proximate event such as recent travel in MT MdDS to aid in diagnosis. Therefore, the longer duration of non-MT MdDS reported here might be influenced by a potentially longer time to diagnosis and treatment. This potential factor was not specifically assessed in this study, since many of the patients presented for second opinions. It should be noted, however, that the durations reported in this study are not related to the time to diagnosis. A participant may have been diagnosed at 3 months into the symptoms, but participated in the study after 18 months of symptoms and thus be tabulated with a duration of 18 months if they were continuing to experience symptoms.

A second concern is potential recall bias in that some individuals simply may not remember the relevant motion trigger and thus be misclassified as non-MT MdDS. In order to mitigate against this possibility, we chose 2 h of travel as the criteria for diagnosing non-MT MdDS, since this duration is generally outside the normal range for most people’s daily activities. For MT MdDS participants, the proximate motion trigger was clear, prolonged motion, which was outside their normal daily activities. The onset of non-MT MdDS, however, was usually not as discrete as MT MdDS, with symptoms often evolving over days or weeks. Therefore, though there is some risk to misattributing a particular participant, we do not suspect that this affected many individuals.

It is possible that both MT and non-MT MdDS represent a convergent physiologic state that has been entered by two different pathways. Prior functional imaging studies have shown enhanced metabolism in the left entorhinal cortex and amygdala in individuals with MT-MdDS along with greater functional connectivity between these limbic regions and posterior visual and vestibular areas compared to controls. These findings were in the setting of lower limbic connectivity with prefrontal cortex (45). Entrainment of these limbic regions through periodic sensorimotor and vestibular input may be the critical factor in the pathophysiology in MT cases, whereas reduced higher order regulation of spatially tuned limbic regions might be a greater factor in non-MT MdDS (5, 46, 47).

Some individuals who have been diagnosed with non-MT MdDS or “spontaneous” MdDS or might also meet the criteria of persistent postural perceptual dizziness (PPPD). The PPPD diagnosis was created to encompass the characteristics of chronic subjective dizziness, space, and motion discomfort, and visual vertigo (48, 49). Proposed diagnostic criteria for PPPD include chronic dizziness and unsteadiness of at least three months duration that is of a non-vertiginous (non-rotational) nature that is generally worse on upright stance and which persists throughout the day. Visual stimuli and either active or passive movements exacerbate PPPD. Since some descriptions of PPPD include sensations of rocking, it is not clear whether individuals with non-MT rocking sensations should fall under the umbrella of PPPD vs. MdDS, or how much clinical utility the distinction would afford. Though the effect of passive motion may appear to be a critical difference between PPPD and non-MT MdDS, we did note that 10% of the non-MT participants did report feeling worse with driving, indicating that these disorders fall within a large spectrum.

## Conclusion

We have presented a large body of clinical information on individuals with MT and non-MT MdDS in order to provide a view of the spectrum of these disorders. Given limited treatment choices in MdDS and the need to develop better predictive models for who responds best to certain treatments, a clearer understanding of the basic clinical features and variability in MdDS will help to increase recognition of MdDS in general medical practice, guide therapeutic management, and contribute to more accurate disease categorization.

## Ethics Statement

This study was carried out in accordance with the recommendations of the UCLA School of Medicine Medical IRB #3. The protocol was approved by Medical IRB #3. All subjects gave written informed consent in accordance with the Declaration of Helsinki (26).

## Author Contributions

Conceptualization: Y-HC and RB. Data curation: Y-HC and YC. Analysis: Y-HC and YC. Writing the draft: Y-HC. Reviewing and editing the final draft: Y-HC, YC, and RB.

## Conflict of Interest Statement

The authors report that there were no personal, professional, or financial conflicts of interest in the design and execution of this study.

## Appendix

### Mal de Debarquement Syndrome Symptom Severity Scale

1. No rocking.
2. Barely noticeable rocking. Walking is normal.
3. Mild intermittent rocking that can be easily ignored. Walking is normal.
4. Mild persistent rocking, which is distracting.
5. Moderate intermittent rocking which requires extra attention to balance control. Walking is normal.
6. Moderate persistent rocking that leads to balance difficulty. May occasionally stumble.
7. Moderately severe, but intermittent rocking that impairs walking. Need occasional assistance to walk.
8. Moderately severe persistent rocking that leads to great balance difficulty. Need assistance to walk for greater distances or specific situations.
9. Severe rocking causing great difficulty with walking. Need constant gait assistance.
10. Severe rocking that prevents any walking.
